# Assessing COVID-19 through the lens of health systems’ preparedness: time for a change

**DOI:** 10.1186/s12992-020-00645-5

**Published:** 2020-11-19

**Authors:** Charbel El Bcheraoui, Heide Weishaar, Francisco Pozo-Martin, Johanna Hanefeld

**Affiliations:** Evidence-Based Public Health, Centre for International Health Protection, Robert Koch Institute, Nordufer 20, 13353 Berlin, Germany

**Keywords:** COVID-19, Pandemic, Health system strengthening, Resilience, Development assistance for health

## Abstract

The last months have left no-one in doubt that the COVID-19 pandemic is exerting enormous pressure on health systems around the world, bringing to light the sub-optimal resilience of even those classified as high-performing. This makes us re-think the extent to which we are using the appropriate metrics in evaluating health systems which, in the case of this pandemic, might have masked how unprepared some countries were. It also makes us reflect on the strength of our solidarity as a global community, as we observe that global health protection remains, as this pandemic shows, focused on protecting high income countries from public health threats originating in low and middle income countries. To change this course, and in times like this, all nations should come together under one umbrella to respond to the pandemic by sharing intellectual, human, and material resources. In order to work towards stronger and better prepared health systems, improved and resilience-relevant metrics are needed. Further, a new model of development assistance for health, one that is focused on stronger and more resilient health systems, should be the world’s top priority.

## Background

Not one month into 2020 and only two weeks after having presented the 11 most urgent health challenges for this decade, the World Health Organization (WHO) declared the COVID-19 outbreak as the first global health emergency [1, 2]. As of 6 October 2020, more than 35 million cases and one million deaths related to the virus have been confirmed worldwide [3]. These losses are unacceptable and require us all to re-think the resilience our health systems and our solidarity as a global community: What new lessons have we learned about the resilience of health systems? How could global solidarity contribute to higher levels of health system resilience?

## Health system resilience

Multiple terms are used in the literature when speaking of health system resilience; yet agreement exists that the concept not only relates to a health system’s response to a sudden crisis but to everyday challenges as well [4]. Further, for a health system to be resilient, it has to be locally integrated and grounded. This means that during responses to shocks, the health system needs to be informed by, search for, and draw on local knowledge in terms of local responses [5]. It has been argued that health system actors should (i) embrace the notion of resilience as going beyond responses to sudden shocks, and encompassing everyday resilience, (ii) view health systems as comprised of both system software and hardware and (iii) conceptualize health system resilience as being about creative adaptation and transformation, not simply bouncing back [6]. Following this perspective, three levels of resilience can be applied to health systems: absorptive capacity, adaptive capacity and transformative capacity [7]. In response to the current COVID-19 pandemic, we have seen that even previously high-performing health systems were becoming overstretched to adapt, casting a shadow on their ability to go further and transform [8, 9]. This makes us wonder about the reliability of the metrics we have used so far to rate health systems, how can they be strengthened, and what other metrics that relate specifically to resilience need to be introduced.

## Health care services have, and are still struggling to resile worldwide

The last months have shown that the COVID-19 pandemic is putting enormous pressure on health care services around the world. Overcrowded hospitals, truck convoys taking the dead to rapidly established cemeteries, and exhausted doctors and nurses battling to save lives with scarce resources provide evidence of the insufficient ability of health care services to deal with the outbreak even in high income countries. Modelled estimates suggest that the load on hospital resources in the USA and the European Economic Area (EEA) at the peak of the pandemic’s first wave in April was well beyond the current hospital capacity [10]. At the end of March, the world demand for ventilators was 10 times that of the number of ventilators available [11]. Global shortages of protective personal equipment have led to an editorial in the Lancet pleading for more protection for frontline health care workers [12]. Unfortunately, shortages are not only in material resources but in the human ones as well. To confront large workforce shortages as a result of the pandemic, EEA countries have been scaling up workforce surge capacity by redeploying staff, incorporating medical students, retired, inactive and foreign-trained health professionals and even volunteers [13].

This troubling situation contradicts assessments of country health system emergency preparedness which were published following the 2014 Ebola Outbreak and which claims to identify future infectious disease hot spots [14]. According to this analysis, the vast majority of the least prepared countries are supposedly in Southeast Asia, West Asia, and Central and Western Africa. While these countries are carrying their share of the burden of COVID-19, higher income countries have been the most disproportionately affected in the initial stages of the pandemic based on available data. Further, on the healthcare access and quality index, a composite measure of health systems’ performance ranging from 0 to 100, the United States scores 88.7, Spain 91.9, Italy 94.9, and China 77.9. In comparison most Central and Western African countries score less than 31 but have been so far not as affected by this pandemic as one would expect [15, 16]. Several factors might have caused healthcare systems in high-income countries to be hit harder, besides the more pronounced aging of their populations, an added risk factor for COVID-19 mortality [17]. We know by now that the burden of nom-communicable disease, which is higher in high-income countries, is strongly correlated with the severity and mortality of COVID-19 [18, 19]. COVID-19 severity and mortality are also strongly associated with older age. What we also saw during this pandemic is that in most EEA countries, with the exception of Germany, the implementation of many preventive measures such as testing, tracing and isolating cases were quite delayed in comparison to Asia [20]. Further, health care systems in many European countries such as Spain and Italy, decades on austerity measures have weakened healthcare systems [21].

This makes us re-think the extent to which we are using the appropriate metrics in evaluating health systems which have masked how unprepared – at least in the context of this pandemic – some countries were. It is time to revise and improve the quality of existing metrics and start considering new metrics that do not only reflect a health system’s ability to provide services and its emergency preparedness, but also on its capacity to adapt, absorb and transform.

## Limitations in public health’ surveillance and monitoring

COVID-19 has exposed alarming gaps with regard to infectious disease surveillance in several regions of the world, for example in South Asia [22]. In addition, many countries were initially lacking the necessary equipment to test those with COVID-19 symptoms, resulting in selective and insufficient testing (e.g. of hospitalized patients only) and a failure to test key population groups, including health professionals and care home residents [23]. The lack of tests and consistent and timely detection of early cases means that interventions to control the transmission of the virus are not put into place and that emergency responses are heterogeneous and inconsistent. Hence the importance of strengthening surveillance systems at local levels, starting with the smallest health facilities and increasing their capacity to detect and report any aberrant symptoms in a timely fashion. The inception, formulation, adoption, implementation and evaluation of policies to prevent the transmission of a virus, in particular of appropriate surveillance, testing, contact tracing and quarantine measures, hinges on the availability of adequately resourced and well-coordinated pandemic preparedness services. If these services are unavailable, as is the case in many countries, the consequences can be catastrophic. Indeed, many countries might indicate existing capacity in terms of specific International Health Regulations’ indicator, but these indicators are mostly reported at the national level, masking the disparity at the sub-national one in the more rural areas where outbreaks often emerge and spread quietly first [24]. Once again, we see ourselves confronting the issue of the reliability of health systems metrics. Here we do not necessarily refer to the reliability of what these metrics measure, but to their thoroughness, as well as their granularity both in terms of geography, and human variability.

## Diminished global solidarity might have affected the resilience of health systems

Global solidarity is motivated by shared responsibilities, offers a more symmetrical expression of mutual respect between global citizens, and is the unifying force to building a global society [25]. The weakness we are observing in health systems occurs at the same time of rising uncertainties which are caused by a diminished global solidarity as well as divergent visions of the world leaders. This situation brings to light a slit in the juncture between global and national health, and the disparity between countries’ local and global response to tackling the pandemic. Metrics used to track global health protection often remain, as this pandemic shows, focused on assessing the risk to protecting high income countries from public health threats originating in low- and middle-income countries.

Not to deter from the current pandemic, but let’s consider the case of war-torn Yemen. The country is simultaneously managing an epidemic of H1N1, Cholera, and dengue with less than 50% of its health facilities operating [26]. Given the travel ban imposed on Yemen, and therefore the limited threat to the global community, the country is borderline forgotten from almost any assistance. This situation is highly un-ethical. While no cases might be spreading from Yemen to other nations, the resilience of the country’s health system is undoubtedly destroyed beyond a return point any time soon, and the consequences might equally burden powerful high-income nations for years to come. In these times, reason urges us to remind ourselves, as a global community, that national and global interests are truly the same, and that emerging diseases have not, and will not stop from occurring, meaning the same fate will befall the next country next time round. In times like this, politicizing health and halting financial assistance for health are the opposite of what the response to the pandemic should look like [27]. Resilience of health systems, especially in low- and middle-income countries cannot rely on internal resources alone, and is the driver of development assistance for health [28]. This assistance is only possible within a framework of solidarity where more affluent nations can contribute to strengthening health systems at home and elsewhere. In times like this, all nations should come together under one umbrella to respond to the pandemic by sharing intellectual, human, and material resources.

## How can we change?

Focusing on vertical, disease-specific issues is unsustainable. Instead, we need to acknowledge the primacy of horizontal approaches. Further, it’s time we start re-thinking the direction of learning and capacity building in the health development arena. This pandemic has demonstrated that leading higher income nations have a lot to learn from their lower income partners. Successful response measures might not necessarily be expensive but rather require tailored solutions and engaged communities.

## What are the challenges in following such an approach?

Our call for reshaping global health by focusing on health system strengthening and its North-South direction is not the first of its kind. Indeed, and especially following the 2014 Ebola outbreak, voices have been raised worldwide for adopting such new metrics and strategies appropriate to assessing resilience and strength of health systems. However, this topic has been controversial for decades, and not without a reason:

- First, global health funders want to see immediate results for their support, but also want these results to be sustainable [29, 30]. While vertical programs often show near-immediate improvement in health outcomes, this is not a given with health system strengthening interventions of which the evidence for informing policies remains reportedly weak [31]. For instance, the Gavi health system strengthening grants have often been interrupted due to mismanagement of funds, and have hence achieved little of their intended impact, completely undermining their sustainability [32–34]. Moreover, development assistance for health has plateaued in recent years. Within that, the share of sector-wide approaches and health system strengthening of total development assistance for health has decreased from 20.7% in 1990 to 14.4% in 2018, a 43.8% decrease, a trend well reflected in the epidemics we have witnessed in the last decade [35]. This can also be due to the fact that funders are faced with the inconsistency and sub-optimal reliability of health systems’ metrics. This undermines efforts to assess health system weaknesses in a coherent and consistent manner across all development partners, thus preventing them from aligning and optimizing their varied health system strengthening support. To correct this course, we suggest the evaluation of existing health systems’ metrics in order to make them more reliable, and broaden them to reflect on health systems’ resilience.

- Second, the definitions of health system strengthening, as well as resilience of health systems, as we argued earlier, were still either vague, or in development up to the first half of the last decade [31]. Nevertheless, the last few years have witnessed an increased effort towards defining these concepts [4, 5, 7, 36, 37]. Most importantly, this literature now puts governance and health workforce at the heart of what needs to be strengthened for health systems to be resilient. In terms of governance, increasing coordination capacities can help tackle systems’ fragmentation. The global health community needs to follow these academic debates and extend the health system strengthening dialogue from its historical focus on procurement and quantity to soft skills and quality [6]. Further, and beyond the core building blocks of health systems, it’s crucial to measure how health systems’ responsiveness incorporates stakeholders’ input by engaging the communities served by these health systems. The ongoing Salud Mesoamerica Initiative has shown the feasibility of such an approach in helping health systems achieve even more than the intended results. The initiative focused first on systems’ inputs, but then quickly moved to quality in delivering health services by touching on all building blocks as well as systems’ responsiveness, all this while relying on well-defined, and locally developed metrics [38]. The initiative’s success stemmed from its continuously evolving design, its focus on information-based quality improvement, a regional approach, engagement of stakeholders at all levels, governments’ buy-in, and a results-based aid model [39]. This example shows that health system strengthening interventions can be feasible and effective. We need to learn from, and build on successful examples to strengthen health systems and their resilience.

## Conclusion

This decade has started with one of the largest pandemics. The current situation is surrounded by many uncertainties, but one thing is sure: The world needs to stand in solidarity to build strong and resilient health systems. First, the global health community needs to evaluate its current metrics for health systems based on the lessons we have learned, and are still learning during this pandemic. Doing so will help more reliably assess the world’s preparedness, and ultimately prevent future pandemics from having a disastrous effect similar to COVID-19’s, and contain them as close as possible to their source. Second, a new model of development assistance for health, one that is focused on stronger and more resilient health systems, should be the world’s top priority. For this, a serious effort is needed to identify a universal framework for resilient health systems, one that helps countries take a defined approach to strengthening their health systems.

## Data Availability

Not applicable.

## References

[1] Ghebreyesus TA (2020). Urgent health challenges for the next decade.

[2] Organization WH (2020). Statement on the second meeting of the International Health Regulations (2005) Emergency Committee regarding the outbreak of novel coronavirus (2019-nCoV).

[3] Control ECfDPa (2020). COVID-19 situation update worldwide, as of 28 June 2020: European Centre for Disease Prevention and Control.

[4] Turenne CP, Gautier L, Degroote S, Guillard E, Chabrol F, Ridde V (2019). Conceptual analysis of health systems resilience: a scoping review. Soc Sci Med.

[5] Hanefeld J, Mayhew S, Legido-Quigley H, Martineau F, Karanikolos M, Blanchet K (2018). Towards an understanding of resilience: responding to health systems shocks. Health Policy Plan.

[6] Barasa EW, Cloete K, Gilson L (2017). From bouncing back, to nurturing emergence: reframing the concept of resilience in health systems strengthening. Health Policy Plan.

[7] Blanchet K, Nam SL, Ramalingam B, Pozo-Martin F (2017). Governance and capacity to manage resilience of health systems: towards a new conceptual framework. Int J Health Policy Manag.

[8] Legido-Quigley H, Asgari N, Teo YY, Leung GM, Oshitani H, Fukuda K (2020). Are high-performing health systems resilient against the COVID-19 epidemic?. Lancet..

[9] Legido-Quigley H, Mateos-Garcia JT, Campos VR, Gea-Sanchez M, Muntaner C, McKee M (2020). The resilience of the Spanish health system against the COVID-19 pandemic. Lancet Public Health.

[10] IHME COVID-19 health service utilization forecasting team CJM (2020). Forecasting the impact of the first wave of the COVID-19 pandemic on hospital demand and deaths for the USA and European Economic Area countries.

[11] Jinshan Hong DL (2020). World Ventilator Demand Now 10 Times What’s Available, Says Maker. Bloomberg.

[12] The L (2020). COVID-19: protecting health-care workers. Lancet.

[13] Claudia B, Maier GS, Gemma A (2020). Williams What strategies are countries using to expand health workforce surge capacity to treat COVID-19 patients? : World Health Organization.

[14] Oppenheim B, Gallivan M, Madhav NK, Brown N, Serhiyenko V, Wolfe ND (2019). Assessing global preparedness for the next pandemic: development and application of an epidemic preparedness index. BMJ Glob Health.

[15] Blanton RE, Mock NB, Hiruy HN, Schieffelin JS, Doumbia S, Happi C (2020). African resources and the promise of resilience against COVID-19. Am J Trop Med Hyg.

[16] Access GBDH, Quality C (2018). Measuring performance on the healthcare Access and Quality index for 195 countries and territories and selected subnational locations: a systematic analysis from the global burden of disease study 2016. Lancet.

[17] Ruan QR, Yang K, Wang WX, Jiang LY, Song JX (2020). Clinical predictors of mortality due to COVID-19 based on an analysis of data of 150 patients from Wuhan, China (vol 17, pg 851, 2020). Intens Care Med.

[18] Azarpazhooh MR, Morovatdar N, Avan A, Phan TG, Divani AA, Yassi N (2020). COVID-19 pandemic and burden of non-communicable diseases: an ecological study on data of 185 countries. J Stroke Cerebrovasc Dis.

[19] Murray CJL, Aravkin AY, Zheng P, Abbafati C, Abbas KM, Abbasi-Kangevari M (2020). Global burden of 87 risk factors in 204 countries and territories, 1990-2019: a systematic analysis for the global burden of disease study 2019. Lancet..

[20] Han E, Tan MMJ, Turk E, Sridhar D, Leung GM, Shibuya K (2020). Lessons learnt from easing COVID-19 restrictions: an analysis of countries and regions in Asia Pacific and Europe. Lancet..

[21] Stuckler D, Reeves A, Loopstra R, Karanikolos M, McKee M (2017). Austerity and health: the impact in the UK and Europe. Eur J Public Health.

[22] Bhutta ZA, Basnyat B, Saha S, Laxminarayan R (2020). Covid-19 risks and response in South Asia. BMJ.

[23] Hunter DJ (2020). Covid-19 and the stiff upper lip - The pandemic response in the United Kingdom. N Engl J Med.

[24] Organization WH (2020). International Health Regulations: State Party Annual Report.

[25] Frenk J, Gomez-Dantes O, Moon S (2014). From sovereignty to solidarity: a renewed concept of global health for an era of complex interdependence. Lancet..

[26] Dureab F, Al-Awlaqi S, Jahn A (2020). COVID-19 in Yemen: preparedness measures in a fragile state. Lancet Public Health.

[27] Mahase E (2020). Covid-19: trump halts WHO funding in move labelled “petulant” and “short sighted”. BMJ.

[28] Dieleman JL, Cowling K, Agyepong IA, Alkenbrack S, Bollyky TJ, Bump JB (2019). The G20 and development assistance for health: historical trends and crucial questions to inform a new era. Lancet..

[29] Lies Steurs JO, Delputte S, Verschaeve J. EU Donors and health system strengthening: the love-hate relationship with the Global Fund. Develop Stud Res. 2018;5(S1):6–11.

[30] Fund TG (2019). Building resilient and sustainable Systems for Health (RSSH) information note The Global Fund.

[31] Hafner T, Shiffman J (2013). The emergence of global attention to health systems strengthening. Health Policy Plan.

[32] Dansereau E, Miangotar Y, Squires E, Mimche H, group UND, group I (2017). Challenges to implementing Gavi's health system strengthening support in Chad and Cameroon: results from a mixed-methods evaluation. Glob Health.

[33] El Bcheraoui C, Miangotar Y, Daoud F, Squire E, Mimche H (2018). Advantages and disadvantages of channeling Gavi's health system strengthening funds through health partners, a case study. PLoS One.

[34] Mimche H, Squires E, Miangotar Y, Mokdad A, El Bcheraoui C, Group I (2018). Resource allocation strategies to increase the efficiency and sustainability of Gavi's health system strengthening Grants. Pediatr Infect Dis J.

[35] Evaluation IfHMa (2020). Financing Global Health.

[36] Fridell M, Edwin S, von Schreeb J, Saulnier DD (2020). Health system resilience: what are we talking about? A scoping review mapping characteristics and keywords. Int J Health Policy Manag.

[37] Nuzzo JB, Meyer D, Snyder M, Ravi SJ, Lapascu A, Souleles J (2019). What makes health systems resilient against infectious disease outbreaks and natural hazards? Results from a scoping review. BMC Public Health.

[38] El Bcheraoui C, Kamath AM, Dansereau E, Palmisano EB, Schaefer A, Hernandez B (2018). Results-based aid with lasting effects: sustainability in the Salud Mesoamerica initiative. Glob Health.

[39] El Bcheraoui C, Palmisano EB, Dansereau E, Schaefer A, Woldeab A, Moradi-Lakeh M (2017). Healthy competition drives success in results-based aid: lessons from the Salud Mesoamerica initiative. PLoS One.

